# Bisphosphonates attenuate age‐related muscle decline in *Caenorhabditis elegans*

**DOI:** 10.1002/jcsm.13335

**Published:** 2023-09-18

**Authors:** Luke Slade, Shelby E. Bollen, Joseph J. Bass, Bethan E. Phillips, Kenneth Smith, Daniel J. Wilkinson, Nathaniel J. Szewczyk, Philip J. Atherton, Timothy Etheridge

**Affiliations:** ^1^ University of Exeter Medical School Exeter UK; ^2^ Faculty of Health and Life Sciences University of Exeter Exeter UK; ^3^ Centre of Metabolism, Ageing & Physiology (COMAP), MRC‐Versus Arthritis Centre for Musculoskeletal Ageing Research (CMAR), Unit of Injury, Recovery and Inflammation Sciences (IRIS), School of Medicine University of Nottingham Derby UK; ^4^ Ohio Musculoskeletal and Neurological Institute Heritage College of Osteopathic Medicine Athens OH USA

**Keywords:** Healthspan, Lifespan, Muscle, Sarcopenia, Zoledronic acid

## Abstract

**Background:**

Age‐related muscle decline (sarcopenia) associates with numerous health risk factors and poor quality of life. Drugs that counter sarcopenia without harmful side effects are lacking, and repurposing existing pharmaceuticals could expedite realistic clinical options. Recent studies suggest bisphosphonates promote muscle health; however, the efficacy of bisphosphonates as an anti‐sarcopenic therapy is currently unclear.

**Methods:**

Using *Caenorhabditis elegans* as a sarcopenia model, we treated animals with 100 nM, 1, 10, 100 and 500 μM zoledronic acid (ZA) and assessed lifespan and healthspan (movement rates) using a microfluidic chip device. The effects of ZA on sarcopenia were examined using GFP‐tagged myofibres or mitochondria at days 0, 4 and 6 post‐adulthood. Mechanisms of ZA‐mediated healthspan extension were determined using combined ZA and targeted RNAi gene knockdown across the life‐course.

**Results:**

We found 100 nM and 1 μM ZA increased lifespan (*P* < 0.001) and healthspan [954 ± 53 (100 nM) and 963 ± 48 (1 μM) vs. 834 ± 59% (untreated) population activity AUC, *P* < 0.05]. 10 μM ZA shortened lifespan (*P* < 0.0001) but not healthspan (758.9 ± 37 vs. 834 ± 59, *P* > 0.05), whereas 100 and 500 μM ZA were larval lethal. ZA (1 μM) significantly improved myofibrillar structure on days 4 and 6 post‐adulthood (83 and 71% well‐organized myofibres, respectively, vs. 56 and 34% controls, *P* < 0.0001) and increased well‐networked mitochondria at day 6 (47 vs. 16% in controls, *P* < 0.01). Genes required for ZA‐mediated healthspan extension included *fdps‐1*/FDPS‐1 (278 ± 9 vs. 894 ± 17% population activity AUC in knockdown + 1 μM ZA vs. untreated controls, respectively, *P* < 0.0001), *daf‐16*/FOXO (680 ± 16 vs. 894 ± 17%, *P* < 0.01) and *agxt‐2*/BAIBA (531 ± 23 vs. 552 ± 8%, *P* > 0.05). Life/healthspan was extended through knockdown of *igdb‐1*/FNDC5 (635 ± 10 vs. 523 ± 10% population activity AUC in gene knockdown vs. untreated controls, *P* < 0.01) and *sir‐2.3*/SIRT‐4 (586 ± 10 vs. 523 ± 10%, *P* < 0.05), with no synergistic improvements in ZA co‐treatment vs. knockdown alone [651 ± 12 vs. 635 ± 10% (*igdb‐1*/FNDC5) and 583 ± 9 vs. 586 ± 10% (*sir‐2.3*/SIRT‐4), both *P* > 0.05]. Conversely, *let‐756*/FGF21 and *sir‐2.2*/SIRT‐4 were dispensable for ZA‐induced healthspan [630 ± 6 vs. 523 ± 10% population activity AUC in knockdown + 1 μM ZA vs. untreated controls, *P* < 0.01 (*let‐756*/FGF21) and 568 ± 9 vs. 523 ± 10%, *P <* 0.05 (*sir‐2.2*/SIRT‐4)].

**Conclusions:**

Despite lacking an endoskeleton, ZA delays *Caenorhabditis elegans* sarcopenia, which translates to improved neuromuscular function across the life course. Bisphosphonates might, therefore, be an immediately exploitable anti‐sarcopenia therapy.

## Introduction

Age‐related muscle decline, termed ‘sarcopenia’, is classified as a specific disease associated with numerous health risk factors in the elderly, prevalent in at least 10% of older populations^1^ and up to 31% as a co‐morbidity.^2^ Despite associated annual healthcare costs that run into the billions^3^ and significant impacts on individual quality of life and all‐cause mortality,^4^ efficacious treatment strategies against sarcopenia remain limited.

Physical activity plus targeted nutrition represents the most effective chronic modifiable lifestyle strategy against sarcopenia.^5^ However, ageing muscle growth responses to these interventions are sub‐optimal,^6^ and certain older individuals can be unable (i.e., through injury and/or access to exercise facilities) or unwilling to participate in regular vigorous activity. Indeed, ~50–70% of those aged 75+ years are physically inactive,^7^ meaning complementary pharmaceutical therapies are relevant to a significant portion of the ageing population. Established drugs that modify human muscle ageing are limited, and importantly, many of these drugs display variable therapeutic efficacy and associate with harmful side‐effect profiles that render them inappropriate for long‐term clinical use. For example, rapamycin holds variable efficacy against sarcopenia indices across health and disease^8^ but is a potent immunosuppressant^9^ unsuitable for chronic use in otherwise healthy humans. Drug identification efforts might instead focus on repurposing existing clinically approved compounds for sarcopenic indications as a realistic, immediately employable approach to therapeutic discovery.

Bisphosphonates are chemically stable derivatives of endogenous inorganic pyrophosphate, a metabolite of multiple biosynthetic reactions and present in most tissues, including muscle.^10^ Recapitulating the effects of inorganic pyrophosphate, bisphosphonates inhibit calcification and lower hydroxyapatite degradation,^11^ while also promoting osteoclast apoptosis to effectively protect against bone loss. As such, bisphosphonates have become a standard clinical strategy against osteoporosis and a variety of skeletal conditions.^12^ Yet skeletal muscle health and bone function are inextricably linked: Effective mechanical coupling and endocrine muscle‐bone communication is essential to muscle and bone health,^13^ with sarcopenia and osteoporosis often presenting together.^14^ Osteoporosis patients prescribed alendronate or pamidronate (the most popular first‐line bisphosphonates) for between 6 months and 3 years display significant increases in muscle strength^15^ and total muscle mass.^16^ Recent reports on the most potent bisphosphonate, zoledronic acid (ZA), show improved muscle morphology, strength and mass in rodent models of cancer cachexia,^17^ Duchenne muscular dystrophy^18^ and denervation‐induced muscle atrophy.^19^ Retrospective analysis of osteoporosis patients prescribed ZA for 3 years also exhibit significant increases in total lean muscle mass, compared with a loss of muscle mass in untreated controls.^20^ In the ageing context, ZA‐treated fruit flies displayed increased survival and improved climbing ability across the life course, suggesting improved neuromuscular ageing.^21^ However, the direct anti‐sarcopenic efficacy of ZA has not been established.

The nematode *Caenorhabditis elegans* is an established model of ageing, displaying ordered, progressive age‐related deterioration of multiple tissues, including muscle.^22^ *C. elegans* muscle is also highly morphologically and metabolically similar to human muscle and has high genetic orthology (60–85%) of sub‐muscular systems with people.^23^ As with fruit flies, *C. elegans* are invertebrates that do not possess clear orthologues of mammalian bone secretory factors (‘osteokines’).^24^ *C. elegans* are, therefore, an excellent system for understanding muscle‐specific mechanisms of bisphosphonates, independently of bone‐derived confounders. We thus employed *C. elegans* as a genomic model of ageing muscle decline to establish the bone‐independent effects, and mechanisms of, bisphosphonates on sarcopenia progression as part of an ageing drug translational pipeline with realistic, near‐term applicability in the clinic.

## Methods

### Assessing zoledronic acid effects on *Caenorhabditis elegans* lifespan and healthspan

Wild‐type (N2 stain) animals were age synchronized by gravity flotation and ~80 L1 larvae were placed on 33 mm petri dishes (20 L1 per 33 mm plate) containing 2 mL NGM agar [50 mM NaCl, 0.25% (w/v) bacteriological peptone, 1.7% (w/v) agar, 1 mM CaCl_2_, 1 mM MgSO_4_, 25 mM KH_2_PO_4_ (pH 6), 12.9 μM cholesterol] and seeded with 200 μL of OP50 *Escherichia coli* bacteria. For ZA treatments, 100 μL of dilute compound (i.e. – 100 μL of 2.3 μM, 23 μM, 230 μM, 2.3 mM and 11.5 μM intermediate dilutions in ddH_2_O for 100 nM, 1 μM, 10 μM, 100 μM and 500 μM concentrations, respectively) were diluted into a 2.3 mL plate volume to achieve the desired final concentration. Control animals were grown on plates seeded with 100 μL of ddH_2_O to account for any diluting of bacterial lawns. Animals were grown on petri dishes for ~48 h at 20°C to reach young adulthood, then washed off using 3 mL M9 buffer (3 g KH_2_PO_4_, 6 g Na_2_HPO_4_, 5 g NaCl, 1 mL 1 M MgSO_4_ per litre) and pooled into 60 cm petri dishes. From these pooled samples, ~70 young adults were collected and loaded into microfluidic chips (Infinity chips; Nemalife Inc., TX) using a 2.5 mL syringe, where the animals remained throughout the rest of the life course. Using the Infinity screening system (Nemalife Inc., TX), on every day of the life course, chips were washed for 90 s with liquid NGM to remove progeny followed by a further 90 s recording for subsequent computational analysis of animal survival and locomotion. Chips were then injected with ~200 μL of 20 mg/mL OP50 supplemented with ZA or ddH_2_O. Specifically, 980 μL of 20 mg/mL OP50 was supplemented with 20 μL of the required ZA intermediate dilution (i.e., 5 μM, 50 μM and 500 μM intermediates for 100 nM, 1 μM and 10 μM of ZA, respectively) or 20 μL of ddH_2_O for controls. Chips were then stored in petri dishes at 20°C for the next 24 h with a damp, sterile tissue and parafilmed to avoid the microfluidic arena drying out.

Lifespan was performed using the Infinity system screening platform (Nemalife Inc., TX). Specifically, 90 s videos were split into three still image frames (frames 1, 300 and 900), and the Infinity screening system automates a bounding box around every (~70–80) whole animal in the microfluidic chip. Using the Infinity system software (Nemalife Inc., TX), computational coefficients determine animal displacements from outside this bounding box and were used for both lifespan and healthspan analysis. Animals were deemed dead if coefficients were <0.01 (no movement) and alive if values were >0.01. For healthspan analysis, movement rate was employed as one of the most robust measures of animal healthspan.^25^ For quantification, animals that obtained coefficients of 0.01–0.40 (i.e., unable to move more than half its body length between subsequent frames) were deemed inactive. Animals with scores of 0.41–1 were classed as ‘active’ animals (i.e., moving half of, or more than its full body‐length in displacement between subsequent frames).

Both lifespan and healthspan were determined every day using videos of microfluidic‐housed populations. Average values were taken from movement scores between all three frames. In addition to software automation, each video was manually corrected for any false positives/box deformations that could skew movement scores. Daily mean movement values were converted to area under the curve to denote total population movement scores across the entire life course.

### Zoledronic acid effects on sarcopenia dystrophic progression with age

The anti‐sarcopenic effects of ZA (1 μM) was visualized and quantified using transgenic strains expressing green fluorescent protein (GFP)‐tagged sarcomeres (PJ727 strain: *jls01* (myo‐3:: GFP, *rol‐6* (*su1006*)); *unc‐54*::lacZ V) or GFP‐tagged mitochondria (CB5600 strain: *ccIs4251* (Pmyo‐3:: Ngfp‐lacZ; Pmyo‐3:: Mtgfp) I; *him‐8* (e1489) IV). Strains were grown on 33 mm plates containing 2 mL NGM agar seeded with 200 μL of OP50 ± ZA, as detailed above. Animals were transferred onto fresh plates every 2 days after young adult stage to remove progeny and prevent population starvation. To image sarcomere and mitochondrial structure, ~30 animals per condition/time point were picked into 20 μL of M9 buffer on a microscope slide (VWR Superfrost, UK) with a cover slip placed on top and imaged immediately using an upright epiflourescent microscope (BX43, Olympus Life Science, UK). Images of the body wall muscles were captured from the head and tail region of each animal (avoiding the mid‐animal/vulva region that exhibits frequent egg‐laying induced tissue disruption) using an Orca‐spark camera (Hamamatsu, Japan) set to 50 ms exposure rate and a gain of 3.4 dB for all acquired images. Approximately 10 individual muscle cells were scored per animal equating to ~300 muscle cells per condition/replicate and scored as either well networked, moderately fragmented or severely fragmented muscle sarcomere or mitochondria structures as previously described^26^ (see *Figure* S1 for representative images of each class of structural defect). Analysis was carried out in ImageJ, and the number of well networked, moderately fragmented or severely fragmented muscles were expressed as a percentage of total muscle cells visible and analysed within each animal.

### RNA interference and combined zoledronic acid treatments for lifespan and healthspan analysis

The effects of RNAi treatments on animal lifespan/healthspan were determined using bacterial feeding vectors and performed in the Infinity screening system, as described above. All bacterial lawns expressing double‐stranded RNA were grown from the MRC Ahringer Library of bacterial clones to target (*fdps‐1: R06C1.2; daf‐16: R13H8.1; agxt‐2: T09B4.8; igdb‐1: T04A11.3; let‐756: C05D11.4; sir‐2.2: F46G10.7: sir‐2.3: F46G10.3*). Each clone was streaked onto LB plates supplemented with 50 μg/mL ampicillin and 25 μg/mL carbenicillin and grown overnight at 37°C. Single colonies were then picked into sterile LB broth supplemented with 50 μg/mL ampicillin and grown for 16 h with shaking at 180 rpm at 37°C. After 16 h incubation, 0.4 mM isopropyl β‐d‐1‐thiogalactopyranoside was added for RNAi induction, with further shaking at 180 rpm for 2 h at 37°C as described previously.^27^ After 18 h incubation, tubes were spun at 3000 *g* for 10 min to obtain bacterial pellets. Liquid NGM was added to each bacterial pellet to reach a final bacteria concentration of 20 mg/mL, adding isopropyl β‐d‐1‐thiogalactopyranoside to a final 1 mM concentration before use. For combined ZA treatments, the same bacterial preparation was performed, but with 20 μL of dilute ZA supplemented to 1 mL of 20 mg/mL OP50 in NGM for a final concentration of 1 μM. On every day of the life course, after washing/recording using the Infinity system as detailed above, ~200 μL of RNAi bacteria ± ZA (1 μM) was injected into the microfluidic chips before storing in petri dishes at 20°C for 24 h until washing/refeeding the next day (with drug dilution/control). All bacteria and drug solutions were prepared fresh daily until cessation of life. RNAi experiments were always conducted alongside empty vector controls (PL4440).

### Statistics

Lifespan analysis was performed using Kaplan–Meier curves assessed by the log‐rank (Mantell–Cox) test. Multiple comparisons of survival curves were performed manually using the Bonferroni‐corrected threshold. For movement rate, area under the curve (AUC) was performed on mean movement values across each day of adulthood (averages taken from across all three frames). AUC values and standard deviations obtained from replicate screens were then assessed by one‐way ANOVA in GraphPad, Prism. Both mitochondrial and sarcomere quantification were analysed by two‐way ANOVA using the percentage classification score from individual animals across biological repeats. All statistics were performed in GraphPad, Prism (San Diego, California USA).

## Results

### Zoledronic acid extends *Caenorhabditis elegans* lifespan and healthspan

**Figure 1:**
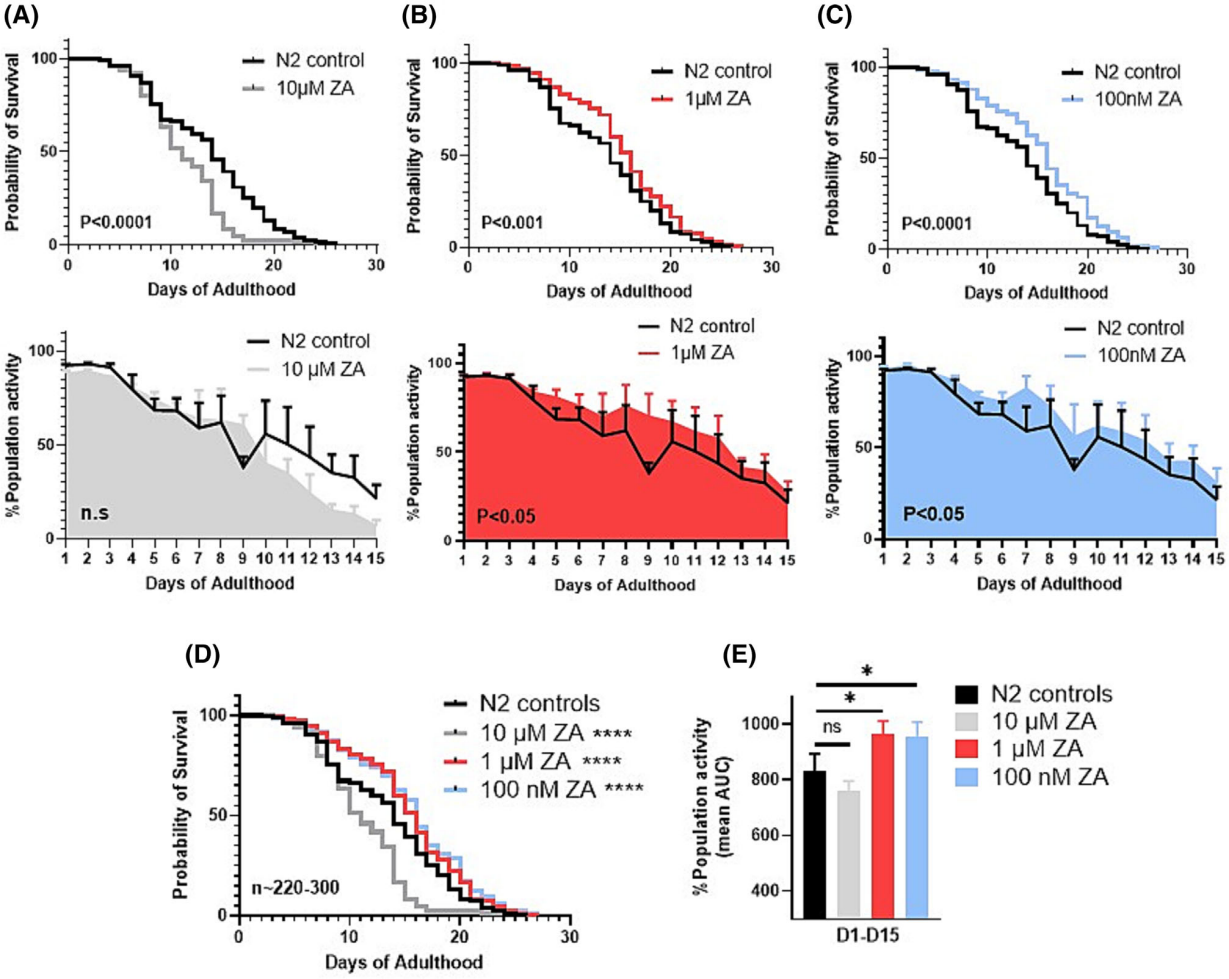
Zoledronic acid acts dose‐dependently to improve *C. elegans* survival and movement capacity. (A) Animals exposed to 10 μM zoledronic acid (ZA) exhibit significant declines in survivability (top) to untreated controls (median survival: 10 vs. 14 days, respectively, *P* < 0.0001), however, show no significant impairment in movement capacity (bottom) across whole life (*P* = 0.208). Conversely, both 1 μM (B) and 100 nM (C) were beneficial to animal survival (1 μM median survival: 16 days, *P* < 0.001; 100 nM: 16 days, *P* < 0.0001), with paralleled improvements in movement capacity throughout the life course. (D) Graphical overlay of lifespan curves from each ZA concentration versus untreated controls. (E) Overlay of life course movement rates between ZA doses displayed as % mean AUC. Data are mean + SEM of three biological repeats, with ~225–300 animals per condition/time point. Asterisks denote significance to N2 controls. *P < 0.05, **P < 0.01, ***P < 0.001, ****P < 0.0001.

Higher ZA concentrations (500 and 100 μM) were larval lethal, and 10 μM ZA shortened lifespan (median survival = 11 vs. 14 days for controls, *P* < 0.0001; *Figure* 1A) without significantly impairing healthspan (animal movement AUC = 758.9 ± 37.08 vs. 833.9 ± 59.91 for controls, *P* > 0.05; *Figure* 1A). Conversely, lifespan extension was observed with lower ZA doses of 1 μM (median survival = 16 days, *P* < 0.001; *Figure* 1B) and 100 nM (median survival = 16 days, *P* < 0.0001; *Figure* 1C). These lifespan‐promoting ZA doses also associated with healthspan extension at 1 μM (AUC = 963.1 ± 48.74, *P* < 0.05; *Figure* 1B) and 100 nM (AUC = 953.8 ± 53.77, *P* < 0.05; *Figure* 1C) treatments. Additionally, significant movement decline onset became apparent in day 9 control animals (*P* > 0.01 vs. day 1 adults), which was upheld across all ZA treatment doses at this single time point (+22.2, +31.8 and +17.6% population activity on day 9 vs. untreated controls in 10 μM, 1 μM and 100 nM, respectively). ZA thus acts dose‐dependently to augment animal longevity and health across the life course.

### Zoledronic acid delays age‐related declines in muscle sarcomere and mitochondrial integrity

**Figure 2:**
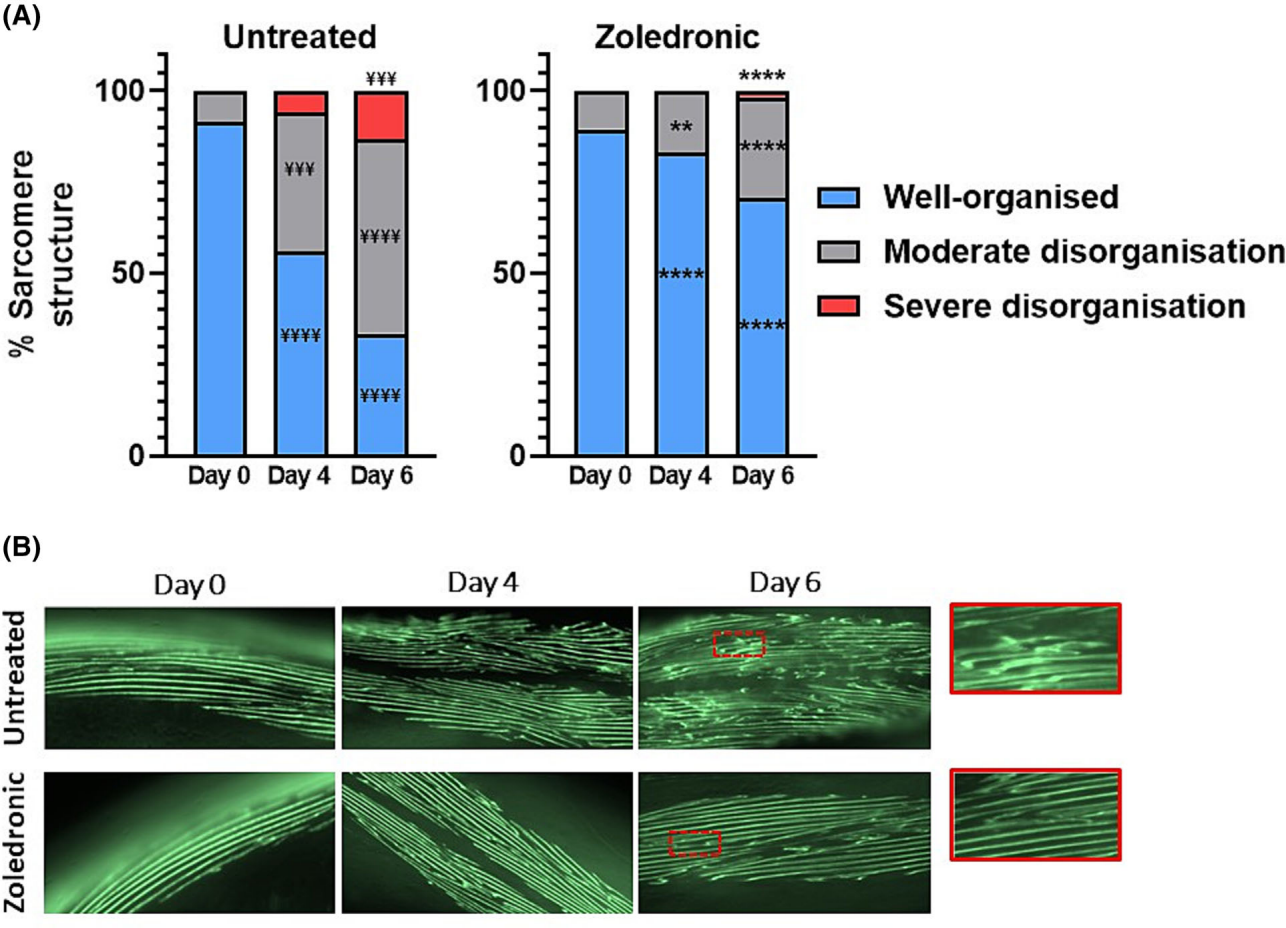
Zoledronic acid attenuates age‐related loss of sarcomere integrity. (A) Onset of myofibrillar disorganization occurred at days 4 and 6 post‐adulthood in untreated animals and was significantly attenuated in zoledronic treated (1 μM) animals (all *P* < 0.0001). (B) Representative images of untreated (top) and zoledronic acid treated (bottom) myofibres. Data are means of two biological repeats, with ~50–60 animals per condition/time point, and images taken from head and tail regions of each animal (~120–150 muscle cells per condition/time point). Asterisks denote significance between zoledronic acid and untreated animals within each structural category (^**^ *P* < 0.01, ^****^ *P* < 0.0001). ‘¥’ denotes significant loss of myofibrillar structure for within‐condition comparisons versus day 0 values (^¥¥¥^ *P* < 0.001, ^¥¥¥¥^ *P* < 0.0001).

To understand the effects of ZA on progressive sarcomere dystrophy across age, transgenic *C. elegans* expressing GFP‐tagged myosin were treated with 1 μM ZA. Corroborating previous reports on the temporal age‐related loss of sub‐cellular muscle structures,^22^ untreated wild‐type animals displayed a significant −35.6% decline in organized myofibrillar structure by day 4 post‐adulthood (*P* < 0.0001; *Figure* 2A,B), which worsened to −58.0% by day 6 post‐adulthood (*P* < 0.0001). ZA delayed loss of organized sarcomeres until day 6 post‐adulthood (−15.6%, *P* < 0.01), and significantly increased the number of muscle cells displaying organized sarcomeres versus untreated controls at days 4 (83.9% vs. 54.4%) and 6 (71.1% vs. 31.4%) post‐adulthood (both *P* < 0.0001, *Figure* 2A,B). ZA also decreased muscle cells categorized as ‘moderately disorganized’ on days 4 (17.0% vs. 38.2%, *P* < 0.01) and 6 (27.6 vs. 53.4%, *P* < 0.0001) post‐adulthood and reduced the number of ‘severely disorganized’ muscles on day 6 post‐adulthood (1.8% vs. 13.1%, *P* < 0.0001).

**Figure 3:**
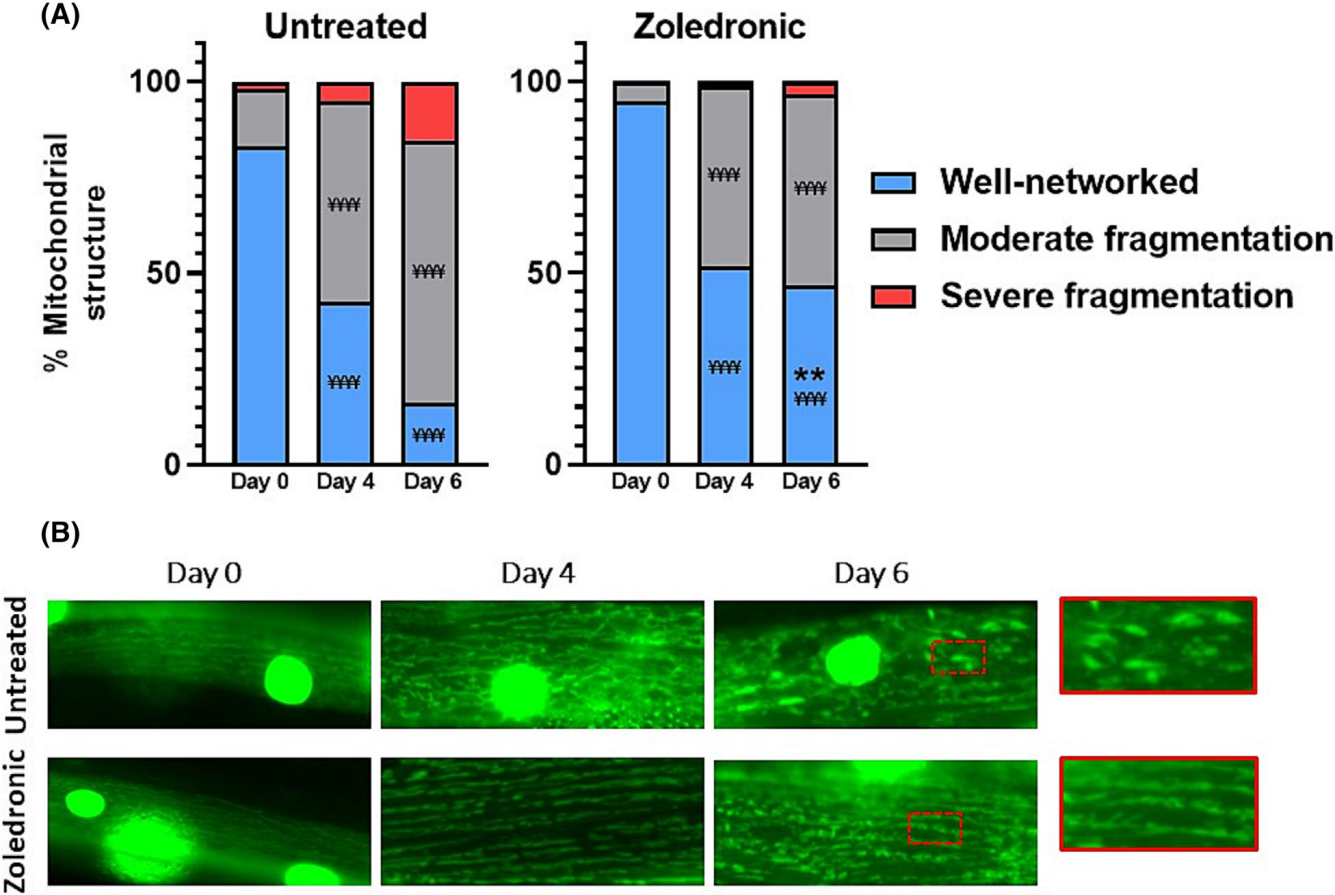
Zoledronic acid attenuates age‐related loss of muscle mitochondrial structure. (A) Untreated animals displayed progressive declines in well‐networked mitochondria across days 0, 4 and 6 post‐adulthood (for each time point, *P* < 0.0001). Zoledronic acid treatment (1 μM) animals saw a similar decline by day 4; however, no further decline was evident by day 6 post‐adulthood, showing significant improvement when compared to untreated animals at this time point (*P* < 0.01). (B) Representative images of untreated (top) and zoledronic acid treated (bottom) data are means of two biological repeats with ~50–60 animals per condition/time point, and images taken from head and tail regions of each animal (~120–150 muscle cells per condition/time point). Asterisks denote significance between zoledronic acid and untreated animals within each structural category (^**^ *P* < 0.01). ‘¥’ denotes significant loss of myofibrillar structure for within‐condition comparisons versus day 0 values (^¥¥¥¥^ *P* < 0.0001).

We next examined the effects of ZA on mitochondrial integrity, given the strong associations of mitochondrial structure and function with ageing animal health. In line with previous wild‐type ageing studies,^22^ loss of networked mitochondria occurred by day 2 post‐adulthood (*Figure* S2), which progressively worsened at day 4 (−42.6%, *P* < 0.0001) and day 6 post‐adulthood (−67.0%, *P* < 0.0001; *Figure* 3A,B). ZA treatment did not delay the onset of well‐networked mitochondria loss at day 2 (*Figure* S2) or day 4 post‐adulthood (51.8%, *P* < 0.0001). However, ZA prevented progressive mitochondrial fragmentation at day 6 post‐adulthood (46.7%, *P* > 0.05 vs. day 4), which was also significantly higher than in untreated animals (46.7 vs. 16.1%, *P* < 0.01).

### Zoledronic acid‐mediated healthspan extension acts through multiple mechanisms

**Figure 4:**
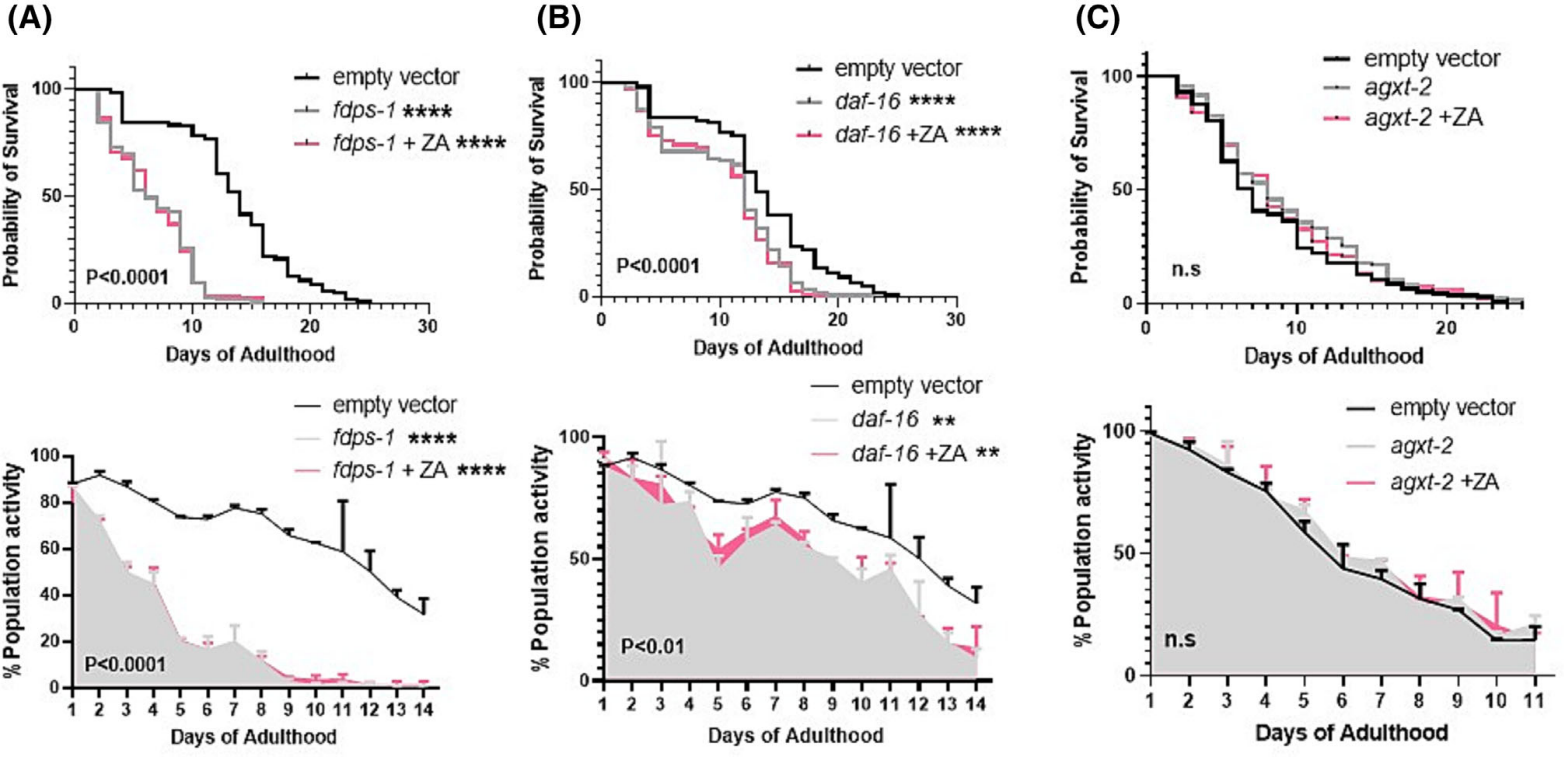
FDPS, FOXO and BAIBA orthologues are required for the healthspan effects of zoledronic acid. Knockdown of *FDPS‐1* (FDPS) with ZA co‐treatment (1 μM) prevents ZA mediated lifespan (top) and healthspan (bottom) extension (A). Similarly, knockdown of *daf‐16* (FOXO) decreases survival and movement, with no improvement with ZA co‐treatment (B). The conserved bone‐muscle ortholog *agxt‐2* (BAIBA) is required for ZA mediated improvement in survival and movement (C). Data are mean + SD of two repeats, with ~140–160 animals per condition/time point. Asterisks denote significance to empty vector. ^**^ *P* < 0.01, ^****^ *P* < 0.0001.

The lifespan extending effects of ZA have previously been attributed to the mevalonate cholesterol/isoprenoid lipid biosynthesis pathway and the FOXO family of transcription factors,^21^ but their role in ZA‐induced healthspan extension is unknown. RNAi knockdown (KD) of *fdps‐1*, an orthologue of farnesyl diphosphate synthetase involved in the biosynthesis of farnesyl diphosphate intermediates from mevalonate, impaired survival (median survival = 6 vs. 14 days for controls, *P* < 0.0001) and healthspan (AUC = 287 ± 9 vs. 894 ± 17% population activity for controls, *P* < 0.0001), which was not improved by co‐treatment with 1 μM ZA (*Figure* 4A). Similarly, RNAi KD of the *daf‐16*/FOXO stress response pathway impaired survivability (median survival = 12 vs. 14 days for controls, *P* < 0.0001) and healthspan (AUC = 680.5 ± 23.6 vs. 894.2 ± 17.4 for empty vector, *P* < 0.01) and prevented the beneficial effects of ZA treatment (*Figure* 4B).

**Figure 5:**
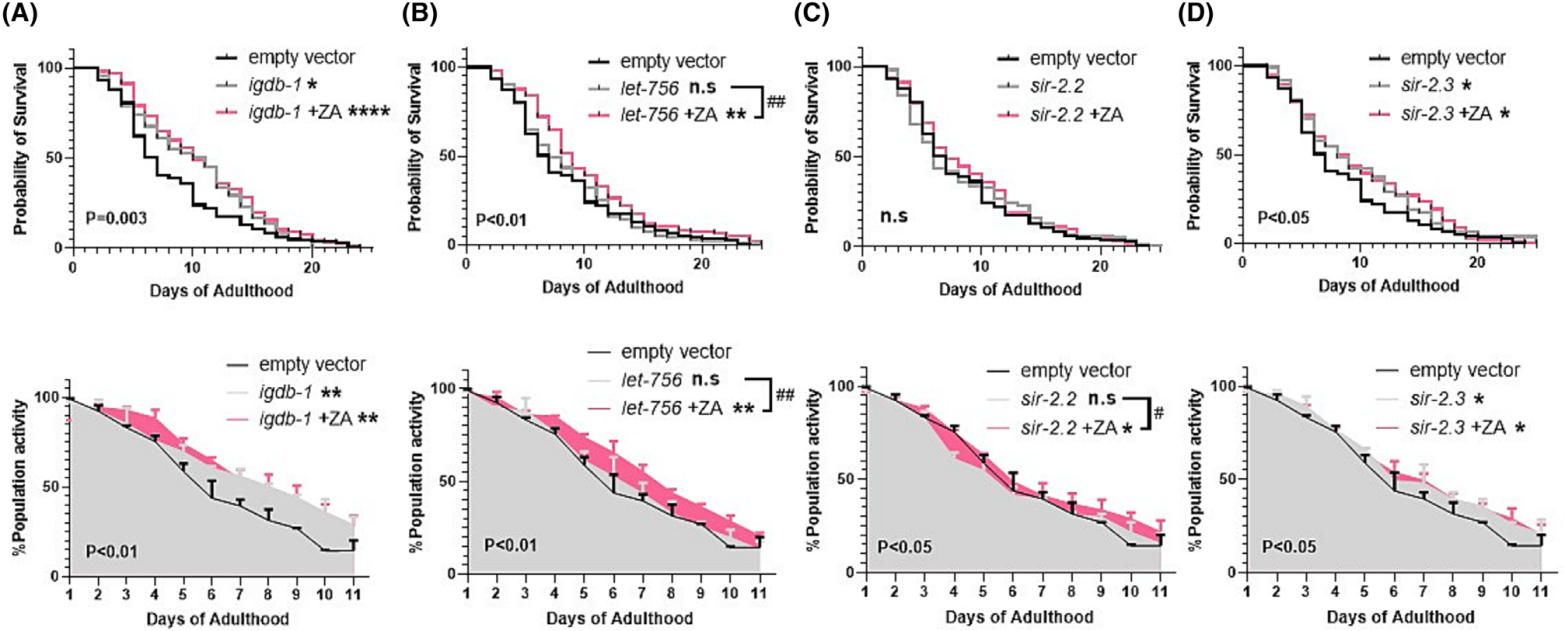
Genes either dispensable, or that associate with ZA's healthspan effects. Knockdown of *igdb‐1* (FNDC5) significantly increased survival and healthspan, where ZA co‐treatment (1 μM) was not synergistic, but displayed similar improvements in lifespan (top) and healthspan (bottom) suggesting *igdb‐1* might exhibit ZA‐induced downregulation (A), or act independently to increase healthspan. Conversely, *let‐756* (FGF21, B) is dispensable for both lifespan and healthspan extension with ZA. The mitochondrial sirtuins displayed opposing effects; *sir‐2.2* (SIRT‐4, C) is dispensable for ZA's healthspan effects, whereas *sir‐2.3* (SIRT‐4) knockdown increased survival and movement (D), as did ZA co‐treatment, suggesting *sir‐2.3* downregulation might mediate ZA's healthspan effects. Asterisks denote significance to empty vector controls. *P < 0.05, ^**^ *P* < 0.01, ^****^ *P* < 0.0001. ‘#’ denote significance between ZA co‐treatments and gene inhibition alone. ^#^ *P* < 0.05, ^##^ *P* < 0.01.

The FOXO pathway is a global stress response system, inducible by a wide array of stimuli and a generic transcription factor regulating cellular responses across multiple drug families^28^ that integrates multiple upstream signals to influence worm ageing.^29^ Alternative, muscle‐centric mechanisms thus also likely contribute to the healthspan benefits of ZA. To probe potential muscle‐derived mechanisms of ZA‐induced healthspan extension, we examined the role of three putative myokines that exhibit altered expression across age and implicated in muscle‐bone crosstalk: *agxt‐2*/BAIBA, *igdb‐1*/FNDC5 and *let‐756*/FGF21.^24^ Lifespan and healthspan were unaffected by KD of *agxt‐2*/BAIBA (β‐aminoisobutyric acid), a muscle‐borne bone protective factor^30^; however, combined *agxt‐2* KD with ZA treatment was unable to confer life/healthspan extension (*Figure* 4C). Conversely, the inhibition of *igdb‐1* extended lifespan (median survival = 11 vs. 7 days for controls, *P* < 0.05) and improved healthspan (AUC = 634.5 ± 10.5 vs. 523 ± 10.0 for controls, *P* < 0.01), with similar life/healthspan extension observed upon co‐treatment with ZA (*Figure* 5A). Additionally, *let‐756* appears dispensable for ZA‐induced healthspan effects: KD alone caused no significant alterations in survival or movement, whereas increased lifespan and healthspan was retained with combined *let‐756* KD and ZA treatment (*Figure* 5B).

Recent rodent work shows bisphosphonates attenuate denervation‐induced muscle atrophy via downregulation of sirtuin‐3 (SIRT3), one of the nicotinamide adenine dinucleotide (NAD+)‐dependent deacetylase family members.^19^ Because SIRT3 is primarily mitochondrially localized,^31^ we examined the role of two *C. elegans* mitochondrial sirtuins, *sir‐2.2* and *sir‐2.3*,^32^ for their role in ZA‐mediated lifespan and healthspan extension. Reinforcing a mitochondrial role of *sir‐2.2* and *sir‐2.3*, RNAi KD of each gene caused increased mitochondrial fragmentation at day 0 post‐adulthood (*Figure* S3). While *sir‐2.2* KD did not alter animal survival or healthspan, loss of *sir‐2.2* blocked ZA's life extending properties but ZA‐induced healthspan extension remained (*Figure* 5C). Additionally, and in line with previous reports in *C. elegans* ,^33^ we find that knockdown of *sir‐2.3* extends lifespan (*Figure* 5D) and healthspan (*Figure* 5H), which remains with combined ZA treatment but is not synergistically beneficial.

## Discussion

Despite the socio‐economic implications, treatment options for sarcopenia remain mostly limited to modifiable lifestyle changes such as diet and exercise, which are incompletely effective.^34^ Complementary drug interventions would, therefore, benefit a significant portion of the ageing population, yet effectual compounds that are safe for long‐term use remain elusive. Identifying drugs in clinical use and repurposing for sarcopenia indications could provide immediately exploitable pharmaceuticals for the general population. Bisphosphonates have been used to safely treat osteoporosis for decades.^35^ Here, we report that bisphosphonates (ZA) extend *C. elegans* lifespan and healthspan, which associates with delayed onset of muscle sarcomere and mitochondrial decline. While general stress response pathways, such as FOXO, regulate these positive healthspan responses to ZA, we establish muscle‐centric myokines (*agxt‐2* and *igdb‐1*) and mitochondrial sirtuin (*sir‐2.3*) as previously unknown regulators of bisphosphonate effects on ageing muscle health.

Growing pre‐clinical^17^, ^18^, ^19^ and human^20^ evidence suggest that bisphosphonates not only preserve bone integrity but also exert muscle promoting effects across a variety of conditions. A recent study in fruit flies suggest similar effects might extend to the ageing process, where bisphosphonate treatment prolonged lifespan and animal healthspan.^21^ We provide the first *in vivo* evidence for a direct role of chronic bisphosphonate administration in delaying age‐related muscle structural decline. Because ageing muscle architectural alterations are a hallmark of human sarcopenia that relate to lowered motility in animals,^22^ bisphosphonate‐mediated sarcomere preservation could translate to reduced sarcopenia in higher animals and people. ZA also attenuated ageing muscle mitochondrial decline. Mitochondrial health is strongly linked with sarcopenic progression across species and mitochondrial structure corresponds with oxidative function^23^; therefore, bisphosphonates might hold dual, and possibly interrelated, efficacy for maintaining muscle metabolic health and sarcomeric integrity. While we assessed animals at, and beyond, the point of onset of age‐related sub‐muscular decline, the anti‐sarcopenic efficacy of bisphosphonates into very old age remains to be determined. Nonetheless, using our microfluidic healthspan device^27^ and animal movement rate as a robust index of health,^25^ we demonstrate that early‐ to mid‐life muscle preservation translates to improved animal health across the entire life course. Bisphosphonates thus represent a promising strategy for maintaining muscle, and whole animal health into older age.

In fruit flies, bisphosphonates increase lifespan through inhibition of the mevalonate metabolic pathway and downstream FOXO activation.^21^ We extend this by implicating the mevalonate‐FOXO system as necessary for bisphosphonate‐mediated health improvements across the life course. The sterol and non‐sterol isoprenoids synthesized via mevalonate localize to plasma membranes and influence several vital cellular processes, the dysfunction of which associate with wide ranging pathologies including cardiovascular disease, autoimmune disease, cancer and Alzheimer's disease.^36^ Our data suggest that sarcopenia can be added to this list, whereby knockdown of a key enzyme in mevalonate metabolism, farnesyl diphosphate synthetase (*fdps‐1*), allows normal larval development to adulthood and young adult (day 1) movement rates, corroborating earlier reports of normal L4 larvae muscle architecture upon *fdps‐1* RNAi.^37^ Thereafter, downregulation of *fdps‐1* causes rapid neuromuscular functional decline (i.e., animal motility), likely indicative of accelerated sarcopenia. Because zoledronic acid is unable to improve lifespan and healthspan in *fdps‐1* or *daf‐16* knockdown animals, the mevalonate‐FOXO pathway is strongly implicated in the ageing neuromuscular health benefits of bisphosphonates.

Bi‐directional communication between muscle and bone has long been established, including mechanical coupling and endocrine muscle‐bone signalling.^13^, ^24^ We, therefore, examined the mechanistic role of three putative muscle‐derived signalling factors (‘myokines’) involved in muscle‐bone crosstalk that exhibit altered age‐related function.^24^ Of these, *agxt‐2*/BAIBA was necessary for the positive healthspan effects of ZA, while *agxt‐2* KD alone was not harmful to animal longevity or health. BAIBA promotes insulin sensitivity, anti‐inflammation^38^ and osteocyte survival in mice,^39^ and its expression does not decline with age^39^; thus, BAIBA‐mediated muscle/health benefits of bisphosphonates would be anticipated to remain in older age. Knockdown of *igdb‐1*/FNDC5 extended worm lifespan and healthspan, countering human studies implicating low circulating FNDC5 as a sarcopenia biomarker.^40^ The healthspan effects of *igdb‐1* KD were also comparable with ZA co‐treatment. This inability of *idgb‐1* KD to neither ablate nor synergistically augment the positive effects of ZA could be interpreted as ZA exerting healthspan‐promoting inhibitory effects on *igdb‐1* or, alternatively, acting through *igdb‐1* independent pathways. Conversely, *let‐756*/FGF21 appears dispensable for ZA‐induced lifespan and healthspan extension. Because *C. elegans* lack an endoskeleton, these findings espouse *agxt‐2*/BAIBA and *igdb‐1*/FNDC5 as muscle‐intrinsic, bone‐independent mechanisms of bisphosphonate action on improving sarcopenia.

Recent rodent work showed that bisphosphonates attenuate denervation atrophy by down‐regulating sirtuin‐3 (SIRT3),^19^ a mitochondrially localized NAD+‐dependent deacetylase with important roles in regulating skeletal muscle metabolism. We observed that knocking‐down a *C. elegans* mitochondrial sirtuin, *sir‐2.3*, improved life‐ and healthspan, whereas mitochondrial *sir‐2.2* KD had no effect, as reported by others.^33^ Because *sir‐2.3* KD with combined ZA was neither inhibitory nor synergistically beneficial to lifespan or healthspan, this supports the model of bisphosphonate‐mediated mitochondrial sirtuin inhibition as a regulator of mammalian muscle health^19^ and now sarcopenia. Loss of *sir‐2.2* was, however, dispensable for the positive effects of ZA. Along with improved mitochondrial structure observed herein, growing evidence thus suggests that bisphosphonates act at least partially through mitochondrial mechanisms to lessen the effects of sarcopenia.

In conclusion, chronic bisphosphonate administration delays the onset of muscle structural and mitochondrial decline in a *C. elegans* model of sarcopenia, and this translates to improved animal health across the entire life course. The mechanisms regulating the positive ageing/muscle effects of bisphosphonates appear to be conserved across species and include the mevalonate‐FOXO metabolic axis, mitochondrial sirtuins and muscle‐derived cytokines. Because worms lack an endoskeleton, these mechanisms of bisphosphonates can also be muscle‐intrinsic. By directly implicating bisphosphonates in attenuating the consequences of sarcopenia, this study adds to a growing body of evidence that bisphosphonates are a clinically safe, immediately exploitable anti‐sarcopenia therapeutic.

## Conflict of interest

The authors declare no competing interests.

## Supporting information


**Figure S1.** Representative images for muscle (*myo‐3*::GFP) and mitochondrial (*mito*::GFP) structural classifications. All images that compose data from figures 2 and 3 (and supplemental figures 1 and 2) were scored into three categories in accordance with the above representative images.

**Figure S2.** Mitochondrial decline in day 2 adults. Both untreated and ZA treated (1 μM) animals display significant mitochondrial decline by day 2 post‐adulthood, and is not restored with ZA treatments. ¥ denotes significant loss of myofibrillar structure for within‐condition comparisons *vs*. day 0 values (¥¥¥ *P* < 0.001, ¥¥¥¥ *P* < 0.0001).

**Figure S3.** Mitochondrial structure with *sir‐2.2* and *sir‐2.3* RNAi. Both *sir‐2.2* and *sir‐2.3* display mitochondrial integrity deficits as early as day 0 of adulthood, supporting their role as mitochondrial sirtuins.
