# Supplementary figures and images for: Bisphosphonates attenuate age‐related muscle decline in Caenorhabditis elegans
Source: J Cachexia Sarcopenia Muscle. 2023 Sep 18;14(6):2613–22. doi: 10.1002/jcsm.13335 (PMC10751425; doi:10.1002/jcsm.13335)

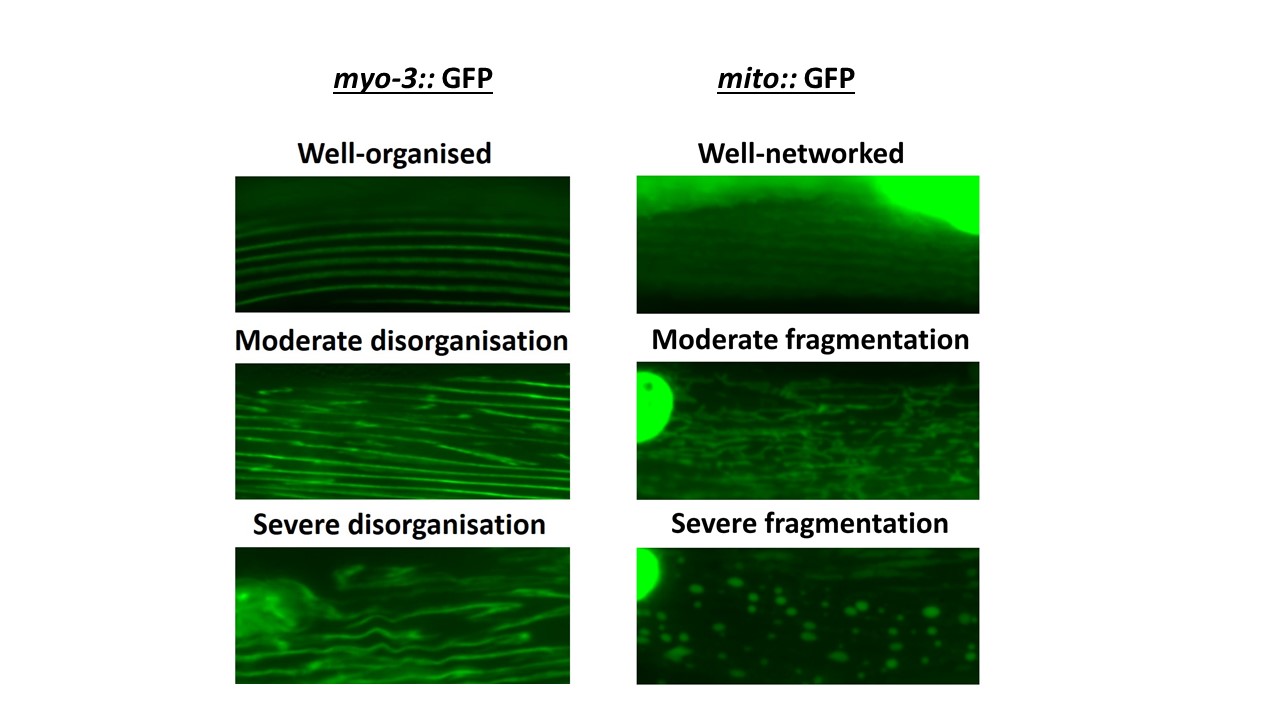

Supplement: Supplementary file 1 — Figure S1. Representative images for muscle (myo‐3::GFP) and mitochondrial (mito::GFP) structural classifications. All images that compose data from figures 2 and 3 (and supplemental figures 1 and 2) were scored into three categories in accordance with the above representative images. [file JCSM-14-2613-s001.jpg]

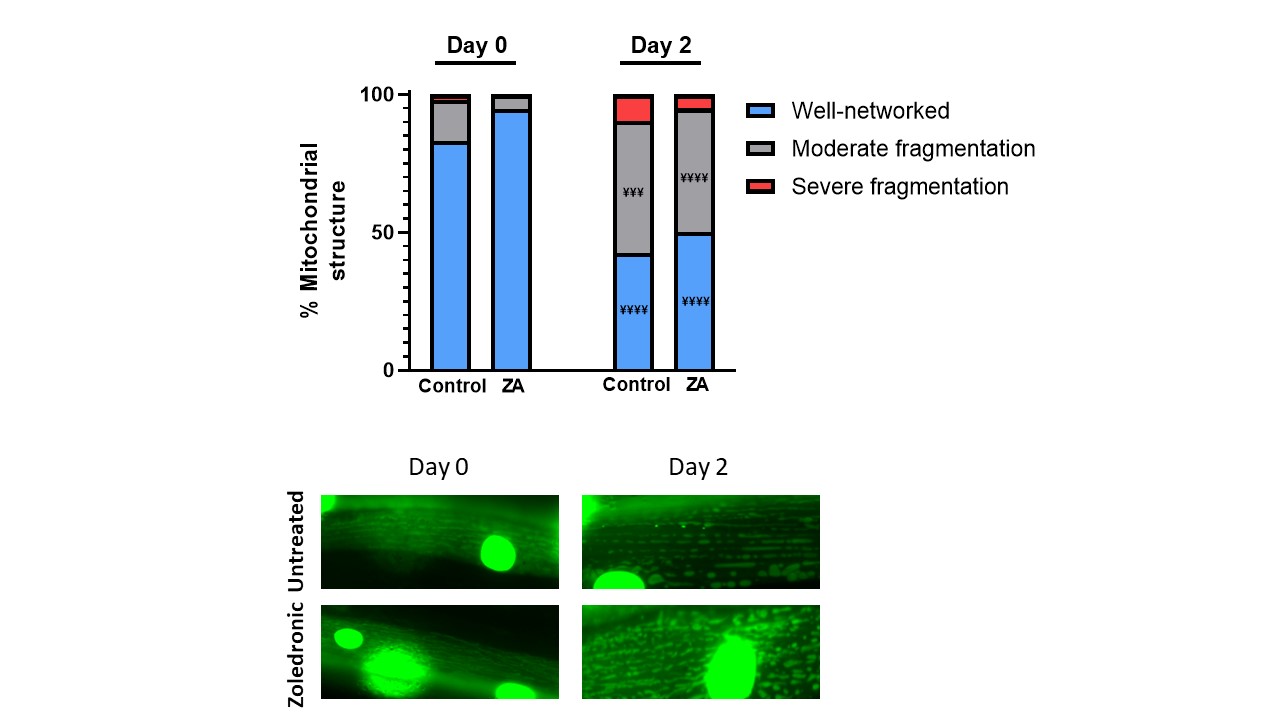

Supplement: Supplementary file 2 — Figure S2. Mitochondrial decline in day 2 adults. Both untreated and ZA treated (1 μM) animals display significant mitochondrial decline by day 2 post‐adulthood, and is not restored with ZA treatments. ¥ denotes significant loss of myofibrillar structure for within‐condition comparisons vs. day 0 values (¥¥¥ P < 0.001, ¥¥¥¥ P < 0.0001). [file JCSM-14-2613-s002.jpg]

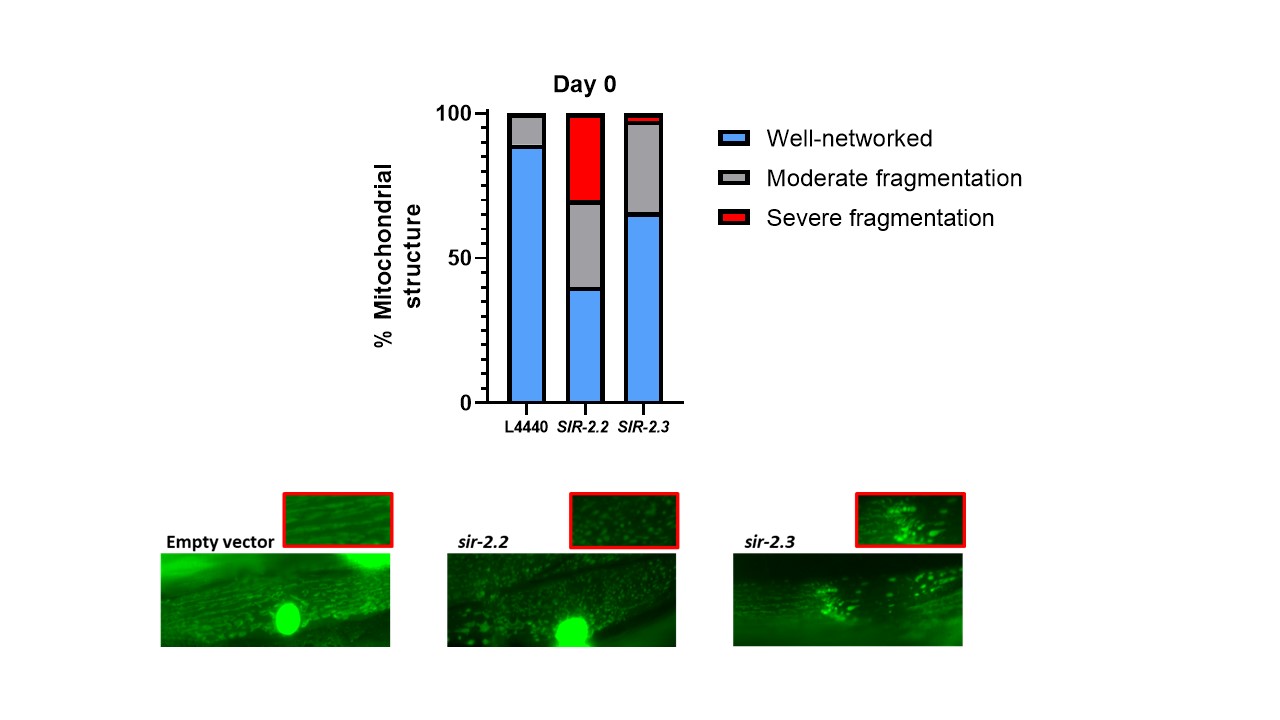

Supplement: Supplementary file 3 — Figure S3. Mitochondrial structure with sir‐2.2 and sir‐2.3 RNAi. Both sir‐2.2 and sir‐2.3 display mitochondrial integrity deficits as early as day 0 of adulthood, supporting their role as mitochondrial sirtuins. [file JCSM-14-2613-s003.jpg]
